# Unraveling the mechanobiology of cornea: From bench side to the clinic

**DOI:** 10.3389/fbioe.2022.953590

**Published:** 2022-10-03

**Authors:** Shu Yang, Jing Zhang, Youhua Tan, Yan Wang

**Affiliations:** ^1^ Clinical College of Ophthalmology, Tianjin Medical University, Tianjin, China; ^2^ Tianjin Eye Institute, Tianjin Key Lab of Ophthalmology and Visual Science, Tianjin Eye Hospital, Tianjin, China; ^3^ Department of Ophthalmology, The First People’s Hospital of Huzhou, Huzhou, Zhejiang, China; ^4^ School of Optometry, Hong Kong Polytechnic University, Hong Kong SAR, China; ^5^ Hong Kong Polytechnic University Shenzhen Research Institute, Shenzhen, China; ^6^ Department of Biomedical Engineering, Hong Kong Polytechnic University, Hong Kong SAR, China

**Keywords:** cornea, mechanobiology, mechanotransduction, mechanical cues, corneal diseases

## Abstract

The cornea is a transparent, dome-shaped structure on the front part of the eye that serves as a major optic element and a protector from the external environment. Recent evidence shows aberrant alterations of the corneal mechano-environment in development and progression of various corneal diseases. It is, thus, critical to understand how corneal cells sense and respond to mechanical signals in physiological and pathological conditions. In this review, we summarize the corneal mechano-environment and discuss the impact of these mechanical cues on cellular functions from the bench side (in a laboratory research setting). From a clinical perspective, we comprehensively review the mechanical changes of corneal tissue in several cornea-related diseases, including keratoconus, myopia, and keratectasia, following refractive surgery. The findings from the bench side and clinic underscore the involvement of mechanical cues in corneal disorders, which may open a new avenue for development of novel therapeutic strategies by targeting corneal mechanics.

## Introduction

**FIGURE 1:**
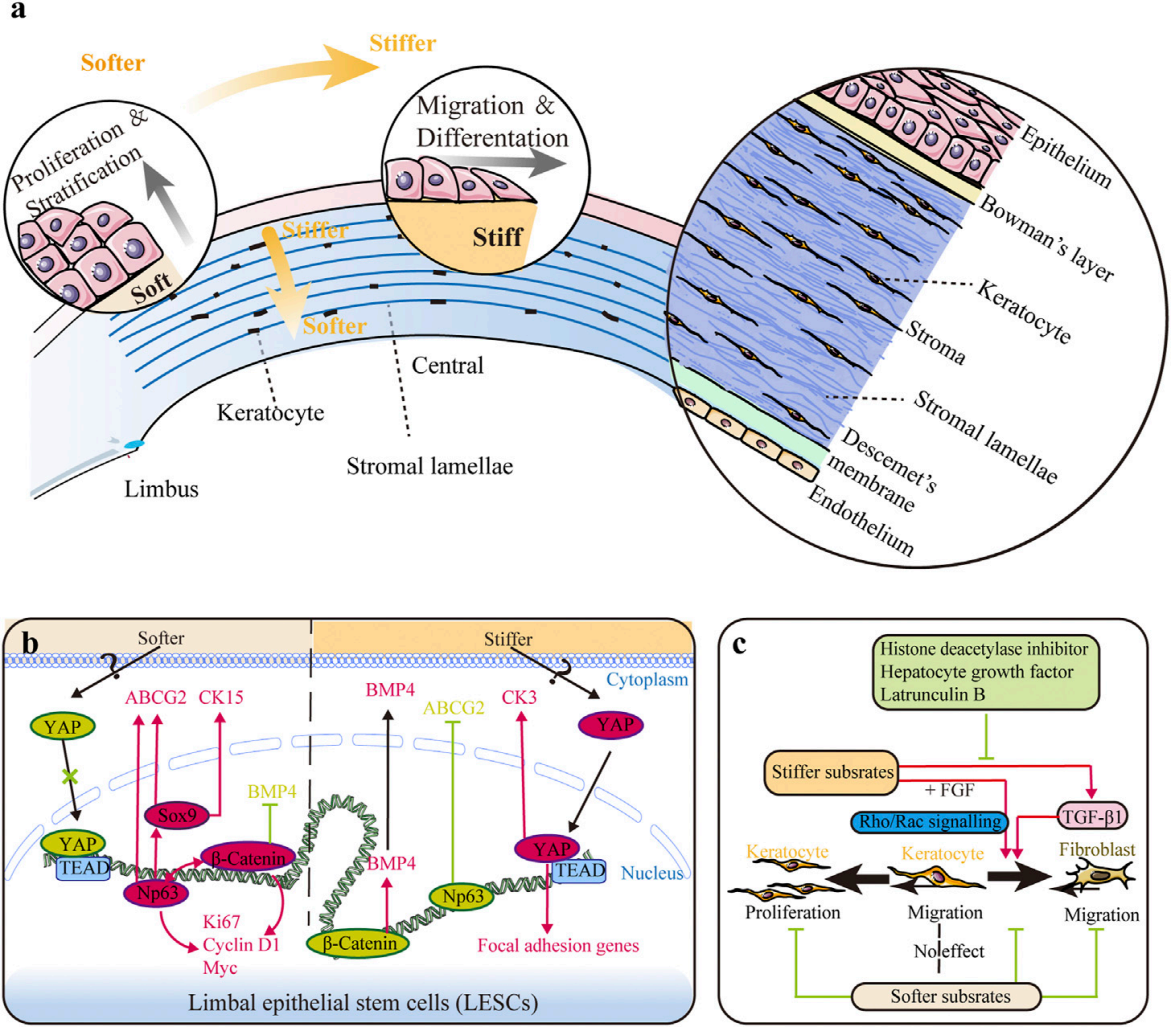
Impact of substrate stiffness. **(A)** Schematic representation of the main changes in substrate stiffness and their effects on the corneal epithelium. The central region is stiffer than the peripheral region in both the corneal epithelium and stroma, and the anterior stroma is stiffer than the posterior stroma. Stiffer substrates promote differentiation of limbal epithelial stem cells (LESCs), while softer substrates promote the proliferation process. **(B)** Substrate stiffness affects the behavior of LESCs *via* the YAP-dependent mechanotransduction pathway with involvement of ΔNp63 and β-catenin. **(C)** Substrate stiffness and chemical factors influence the behaviors of keratocytes. Softer substrates inhibit proliferation of keratocytes and migration of fibroblasts. Softer substrates also preserve the phenotype of keratocytes, while stiffer substrate promotes keratocyte–fibroblast–myofibroblast (KFM) transformation induced by transforming growth factor-β1 (TGF-β1). This stiffness-related transformation could be suppressed by histone deacetylase inhibitors, hepatocyte growth factor, and latrunculin B. Furthermore, extracellular matrix (ECM) stiffness also affects the response of fibroblasts to fibroblast growth factor (FGF) by the interplay between Rho and Rac signaling.

The cornea is the outermost transparent connective tissue of an eye and primarily consists of three layers with different cells: an anterior layer with epithelial cells, a middle stromal layer with abundant extracellular matrix (ECM) and keratocytes, and a posterior layer with endothelial cells (Figure 1A). As a load-bearing tissue of the eye, the cornea is constantly subjected to multiple mechanical cues, such as forces from the eyelid (Shaw et al., 2010), tear film (Jones et al., 2008), aqueous humor (Qin et al., 2021), and intraocular pressure (IOP) (Kwok et al., 2021), and even external forces with possible harmful effects, including eye rubbing, contact lens wearing, and surgical intervention.

Corneal cells can sense and respond to mechanical cues such as substrate topography and stiffness (Then et al., 2011), shear stress (Ren and Wilson 1997; Kang et al., 2014; Duan et al., 2021), and tensile and compressive forces (Liu et al., 2014; Feng et al., 2016; Du et al., 2017; Zhang et al., 2021). Different types of cells in the cornea can perceive and transduce mechanical signals in distinct ways, which modulate the expressions of specific genes and influence diverse biological functions. Recent studies have shown that several corneal diseases, including keratoconus (Amit et al., 2020; Dou et al., 2022), keratectasia (Dupps and Wilson 2006), dry eye disease (Yoshioka et al., 2015; Yamaguchi and Shiraishi 2018), myopia (Kang et al., 2018; Xin et al., 2021), and bullous keratopathy after laser iridotomy (Kaji et al., 2005; Yamamoto et al., 2010), are closely related to abnormal responses of corneal cells to mechanical forces.

In this review, we summarize recent progress made in understanding the ways that corneal cells interact with different mechanical microenvironments to achieve several major biological functions (with a focus on the mechanotransduction process). We then discuss abnormal mechanical alterations of the corneal tissue in various diseases and subsequent effects on corneal cells, which enables physicians to understand pathological mechanisms and develop therapeutic strategies for corneal disorders from a mechanobiological perspective.

## Corneal structure and functions

The cornea consists of five different layers—the epithelium, Bowman’s layer, stroma, Descemet’s membrane, and endothelium—from the anterior to the posterior cornea (Figure 1A). As the outermost anterior part of the cornea, the corneal epithelium is covered by tear fluid and consists of 4–6 layers of nonkeratinized and stratified squamous epithelial cells with various junctional complexes to prevent the passage of external agents into deeper layers of the cornea (Eghrari, 2015). Limbal epithelial stem cells (LESCs) can proliferate in an orderly manner and differentiate to replenish corneal epithelial cells (CEpCs) lost in normal or damaged tissue, thus maintaining the normal layered structure and homeostasis of the corneal epithelium (Secker and Daniels 2008).

Posterior to the epithelial basement membrane is an 8–12 μm acellular, non-regenerating layer called Bowman’s layer (Eghrari, 2015). It is characterized by random arrangement of collagen fibrils and proteoglycans and is originally considered an important stabilizer of corneal curvature (Ma et al., 2018). However, recent studies have suggested that the presence of Bowman’s layer makes a negligible contribution to the entire corneal biomechanics (Torres-Netto et al., 2021).

The stroma, which constitutes up to 90% of corneal thickness, is the main determinant of corneal biomechanics. It comprises approximately 250 (central cornea) to 500 (peripheral cornea) stacked and interweaving collagen lamellae and a sparse population of keratocytes (Meek and Knupp 2015). The aligned collagen fibrils have diameters less than the wavelength of light, and collagen fiber lamellae are approximately 10–200 µm wide and only 1–2.5 µm thick (Meek and Knupp 2015). The lamellae exhibit a preferred orthogonal collagen alignment in the nasal–temporal and superior–inferior orientations within the central cornea, and the fibers tend to run circumferentially within the peripheral cornea (Meek and Knupp 2015). Moreover, the anterior stroma comprises a denser collagen distribution and highly interwoven of lamellae, inserted into Bowman’s layer, which results in relatively stiffer mechanical properties (Meek and Knupp 2015). Type I collagen is the predominant component of collagen fibrils, while type V collagen and small leucine-rich proteoglycans (e.g., lumican and keratocan) potentially modulate collagen-fibril assembly (Massoudi et al., 2016). Biomechanical properties of the stroma are highly dependent on these organizations and interactions among fibrils within different lamellae. Keratocytes are quiescent in the normal cornea and responsible for slow turnover of the stroma. Upon corneal injury, keratocytes adjacent to the injury differentiate into proliferative and metabolically active fibroblasts and subsequently myofibroblasts, which produce greater amounts of collagen, proteases, and cytokines to remodel the arrangement of collagen fibrils in the stroma (Stramer et al., 2003; Chen et al., 2015).

Descemet’s membrane is the basement membrane of corneal endothelial cells (CEnCs) and contains types IV and VIII collagen, laminin, and fibronectin (Eghrari, 2015). It forms a hexagonal lattice, gradually increases in thickness from birth (3 μm) to adulthood (8–10 μm), and maintains corneal relative dehydration.

Finally, the corneal endothelium is in direct contact with the aqueous humor and comprises a monolayer of polygonal, predominantly hexagonal CEnCs with tight junctions and adherens junctions. Its ion-transport system associated with Na^+^/K^+^-ATPase and bicarbonate-dependent Mg^2+^-ATPase counteracts water imbibition into the stroma (Eghrari, 2015). CEnCs cannot proliferate in humans, and loss of or damage to these interconnected CEnCs results in increased water imbibition. In young adults, the endothelial cell density (ECD) within a healthy cornea is approximately 3,000–4,000 cells/mm^2^. However, an abnormal cornea loses its ability to pump sufficient water to maintain its function when ECD decreases below 750–500 cells/mm^2^ (Ramirez-Garcia et al., 2018).

## Principles of mechanosensing and mechanotransduction

Mechanical signals influence cell behavior during tissue homeostasis and in pathological conditions mainly through mechanotransduction. A cell senses mechanical forces through mechanosensors on the cell surface, such as integrins and cadherin’s mechanosensitive ion channels (Ingber 2006). Integrin-mediated adhesion, also known as focal adhesions, can perceive and transfer mechanical cues from the ECM to the cytoskeleton (Sun et al., 2016; Astudillo 2020). This integrin-mediated mechano-transduction relies on several linker proteins (e.g., talin, vinculin), activating downstream signaling molecules, such as focal adhesion kinase, Src, phosphoinositide 3-kinase, YAP/TAZ, myocardin-related transcription factor (MRTF), and serum response factor (SRF) (Sun et al., 2016; Astudillo 2020). Physical stimulus propagation from the ECM to the nucleus might take up to ∼1 ms (Martino et al., 2018). Cadherin-mediated adhesion, also known as adherens junctions, mediates force transduction between cells through several critical signaling molecules, such as cadherin, β-catenin, α-catenin, p120-catenin, vinculin, and zyxin (Ravasio et al., 2022). It can mediate force-induced activation of Ca^2+^ influx through mechanosensitive ion channels and associated actin assembly (Ingber 2006; Ravasio et al., 2022).

Mechanosensors perceive mechanical forces and further transmit them from the cytoskeleton and LINC complex (linker of the nucleoskeleton and cytoskeleton) into the nucleus (Sun et al., 2016; Martino et al., 2018; Astudillo 2020), which has been recognized as a mechanosensor recently (Kirby and Jan 2018). The LINC complex connects the cytoplasmic cytoskeleton with the nuclear lamina through nuclear transmembrane protein emerin and the inner nuclear protein SUN. SUN proteins connect to the lamins that form the lamina and nuclear scaffold, which attach to chromatin and DNA, and influence chromatin organization and gene transcription. Therefore, mechanical forces can directly propagate into the nucleus and regulate mRNA transcription (Tajik et al., 2016). The cytoskeleton provides structural support for cells and bears cellular tension, which is critical in mechanotransduction. Dynamic changes in the components of the cytoskeleton, such as actin fibers (F-actin), microtubules, and intermediate filaments, alter cell mechanical properties. Cytoskeletal tension is closely related to the Rho/ROCK/myosin pathway, which critically regulates actin polymerization and mechanotransduction (Martino et al., 2018). In addition, Yes-associated protein (YAP) and transcriptional coactivator with PDZ-binding motif (TAZ) are potent mechanoresponsive factors, shuttle between the cytoplasm to the nucleus in response to mechanical cues, and regulate transcription of downstream genes (Sun et al., 2016; Astudillo 2020). There are many other mechanosensitive proteins/signaling, such as Wnt, Notch, PI3K/Akt, and MAPK/ERK. In the cornea, only the role of YAP and β-catenin has been identified (Figure 1B).

## The impact of mechanical cues from the extracellular matrix

ECM topography and stiffness have a significant impact on corneal cell behavior (Petroll and Miron-Mendoza 2015; Masterton and Ahearne 2018; Xiong et al., 2019).

### Substrate topography and stiffness

*In vivo*, CEpCs and CEnCs grow on basement membranes, while keratocytes grow between collagen lamellae, which provide unique nanotopographic environments around cells (Figure 1A). *In vitro*, substrate topography (e.g., groove, pillar, or pit patterns) represents the geometrically defined, three-dimensional (3D) environments around cells (Petroll and Miron-Mendoza 2015; Xiong et al., 2019). It can mimic the nanotopographic structures appearing around cells *in vivo* by altering the parameters such as height, depth, width, and spacing of the substrate surface.

Mechanical stiffness is the ability of an object to resist deformation in response to an applied force and is usually represented by elastic modulus. The stiffness of the whole cornea is mainly determined by ECM microstructure and composition, namely, the amount and arrangement of collagen fibrils, the content of proteoglycans, and the hydration and dehydration of tissue (Ruberti et al., 2011). As a heterogeneous tissue, the stiffness of different tissue layers in the human cornea differs a lot (Figure 1A), and the details have been discussed comprehensively in our previous review (Ma et al., 2018). Overall, the central region is stiffer than the peripheral region in both the corneal epithelium and stroma (Mikula et al., 2016; Gouveia et al., 2019a), and the anterior stroma is stiffer than the posterior stroma (Mikula et al., 2016). Bowman’s layer is nearly three times stiffer than the anterior stroma (Last et al., 2012). However, the stiffness of Descemet’s membrane, ranging from kPa to MPa, remains debatable (Ma et al., 2018). Here, substrate stiffness refers to the biomechanical property of the localized ECM that cells experience.

### Substrate stiffness determines the fate of limbal epithelial stem cells

LESC differentiation can be promoted by high substrate stiffness (Figures 1A,B). The expressions of mature epithelial markers (cytokeratin 3 and 12) were found to increase as the biomechanics between the limbus and central cornea tissue shifted from soft (6.24 ± 0.09 and 6.40 ± 0.14 GHz Brillouin frequency shifts in the sub-epithelium and the anterior-most stroma, respectively) to stiff (6.66 ± 0.04 and 6.53 ± 0.04 GHz Brillouin frequency shifts in the sub-epithelium and the anterior-most stroma, respectively) (Jones et al., 2012; Gouveia et al., 2019a; Masterton and Ahearne 2019). It is to be noted that the Brillouin spectro-microscope is utilized to measure the bulk modulus, but not Young’s modulus of the cornea, and thus requires caution during data interpretation (Wu et al., 2018; Yun and Chernyak 2018). High substrate stiffness triggered maturation of LESCs by activating the YAP-dependent mechanotransduction pathway and suppressing ΔNp63 and Wnt/β-catenin signaling and increased the expression of BMP4 (Figure 1B) (Gouveia et al., 2019a; 2019b). Thus, aberrant stiffening of the limbus promotes excessive differentiation of LESCs, thereafter resulting in stem cell deficiency, corneal opacification, and vision loss (Nowell et al., 2016). Collagenase treatment could rescue these alterations by softening the matrix, leading to inactivation of YAP signaling and inhibition of LESC differentiation (Figure 1B) (Gouveia and Connon 2020). This research highlights the potential of regulating LESC function and corneal epithelial tissue regeneration by controlling tissue biomechanics and mechanotransduction.

### Corneal cell growth depends on substrate topography and stiffness

Grooves are the most widely used substrate topography for culturing CEpCs and corneal stromal cells. After culturing on microgrooves or nanogrooves, cells align and elongate along the direction of the groove axis and could be modulated by the depth but not the width of the grooves (Fraser et al., 2008; Xiong et al., 2019). Meanwhile, cytoskeleton fibers and focal adhesions in cells are also aligned along the grooves (Xiong et al., 2019). In contrast, pillar or pit patterns are the most commonly used topographic features in CEnC culture and can mimic the topographical features of hexagonal lattice structures and nanoscale pores on Descemet’s membrane (Last et al., 2009). Both micro- (∼1 µm) and nano-sized (∼250 nm) pillars were found to facilitate the *in vivo*-like morphology of CEnCs, promote their proliferation with higher cell density and smaller cell size (Muhammad et al., 2015; Rizwan et al., 2017), and enhance the expressions of Na^+^/K^+^ ATPase and cell–cell tight junction protein Zonula Occludens-1 (ZO-1) (Koo et al., 2014; Muhammad et al., 2015; Palchesko et al., 2015; Rizwan et al., 2017). Recently, a small patterned hydrogel surface with physiologically relevant hexagon densities (∼2000 hexagons/mm^2^) and a similar elastic modulus to native Descemet’s membrane (∼50 kPa) was constructed and augmented the formation of monolayers with higher cell density (Erkoc-Biradli et al., 2021). In addition, substrates with hexagonal microtopography can promote differentiation of human mesenchymal stem cells into corneal-endothelial-like cells (Gutermuth et al., 2019).

Substrate stiffness can also impact corneal cell growth. Corneal fibroblasts (CFs) align and compact collagen parallel to the axis of the highest ECM stiffness under constrained (anisotropic) conditions, but there is no preferential orientation in the unconstrained (isotropic) ECM (Karamichos et al., 2007). The biomimetic substrate stiffness of CEpCs (∼1.3 KPa) (Molladavoodi et al., 2015), keratocytes (∼25 kPa) (Chen et al., 2020), and CEnCs (∼50 kPa) (Palchesko et al., 2015) can preserve the cytoskeleton structure (actin fibers) and phenotype of corneal cells. Rac1 has been proven to mediate this process in keratocytes (Chen et al., 2020).

### Substrate topography and stiffness facilitate wound healing

Proliferation and migration activities of CEpCs surrounding the wound area facilitate healing of the corneal epithelium (Ljubimov and Saghizadeh 2015). A quiescent keratocyte differentiates into a more proliferative and metabolically active fibroblast, and subsequently a myofibroblast, which is referred to as keratocyte–fibroblast–myofibroblast (KFM) transformation that occurs during the healing of the stroma. This transformation is accompanied by an increased expression of disorganized ECM, collagen-degrading enzymes, and cytokines, resulting in stromal structure remodeling (Hassell and Birk 2010; Ljubimov and Saghizadeh 2015).

Substrates with special patterned topography pitch sizes could prevent the KFM transformation (Pot et al., 2010; Myrna et al., 2012) and limit the migration of CEpCs, fibroblasts, and myofibroblasts only parallel to the grooves (Diehl et al., 2005; Pot et al., 2010). In addition, the proliferation rates of CEpCs and keratocytes on substrates with smaller grooves or groove pitch were inhibited, suggesting that overly narrow grooves may impede cell proliferation (Liliensiek et al., 2006; Xiong et al., 2019). Most importantly, ECM proteins generated by keratocytes cultured on a groove pattern also tended to be parallel to the axis of the grooves, resembling the architecture of a native corneal stroma (Then et al., 2011).

ECM stiffness also affects multiple cellular activities during wound healing. CEpCs displayed a lower migration speed on compliant substrates (Molladavoodi et al., 2015). For stromal cells, ECM stiffness and biochemical cues interact to regulate cell activities (Figure 1C). A compliant microenvironment inhibited proliferation of keratocytes cultured in platelet-derived growth factor BB (PDGF-BB) medium (Iyer et al., 2022). However, reduction of effective ECM stiffness significantly inhibited migration of keratocytes cultured in 10% FBS but had no effect when cultured in PDGF-BB (Kim et al., 2012). Since keratocytes in PDGF maintained a lower level of contractility than those in 10% FBS, substrate stiffness seems to affect migration only under fibroblastic rather than quiescent phenotypes (Kim et al., 2012). Furthermore, low substrate stiffness preserved the phenotype of keratocytes, while high substrate stiffness promoted KFM transformation (Dreier et al., 2013; Chou et al., 2016; Maruri et al., 2020). However, a stiff microenvironment alone is insufficient to induce KFM transformation. The KFM transformation induced by transforming growth factor-β1 (TGF-β1) could be further enhanced on rigid substrates, but this transformation showed no stiffness dependency in the absence of TGF-β1 (Dreier et al., 2013; Maruri et al., 2020). Furthermore, histone deacetylase inhibitors, hepatocyte growth factor, and latrunculin B (an actin cytoskeleton disruptor) could suppress this transformation through their inhibitory effect on α-smooth muscle actin (α-SMA) expression (Koppaka et al., 2015; Miyagi et al., 2018; Thomasy et al., 2018). ECM stiffness also affected the response of CFs to fibroblast growth factor (FGF) and induced stress fiber formation and collagen reorganization, which appeared to be regulated by the interplay between Rho and Rac signaling (Lakshman and Petroll 2012; Petroll and Lakshman 2015). Taken together, these findings demonstrate that substrate topography and stiffness regulate corneal cell behaviors, which may be utilized to maintain the uniform structure of the cornea and impede development of fibrosis and corneal haze in wound healing.

## The impact of shear stress

**FIGURE 2:**
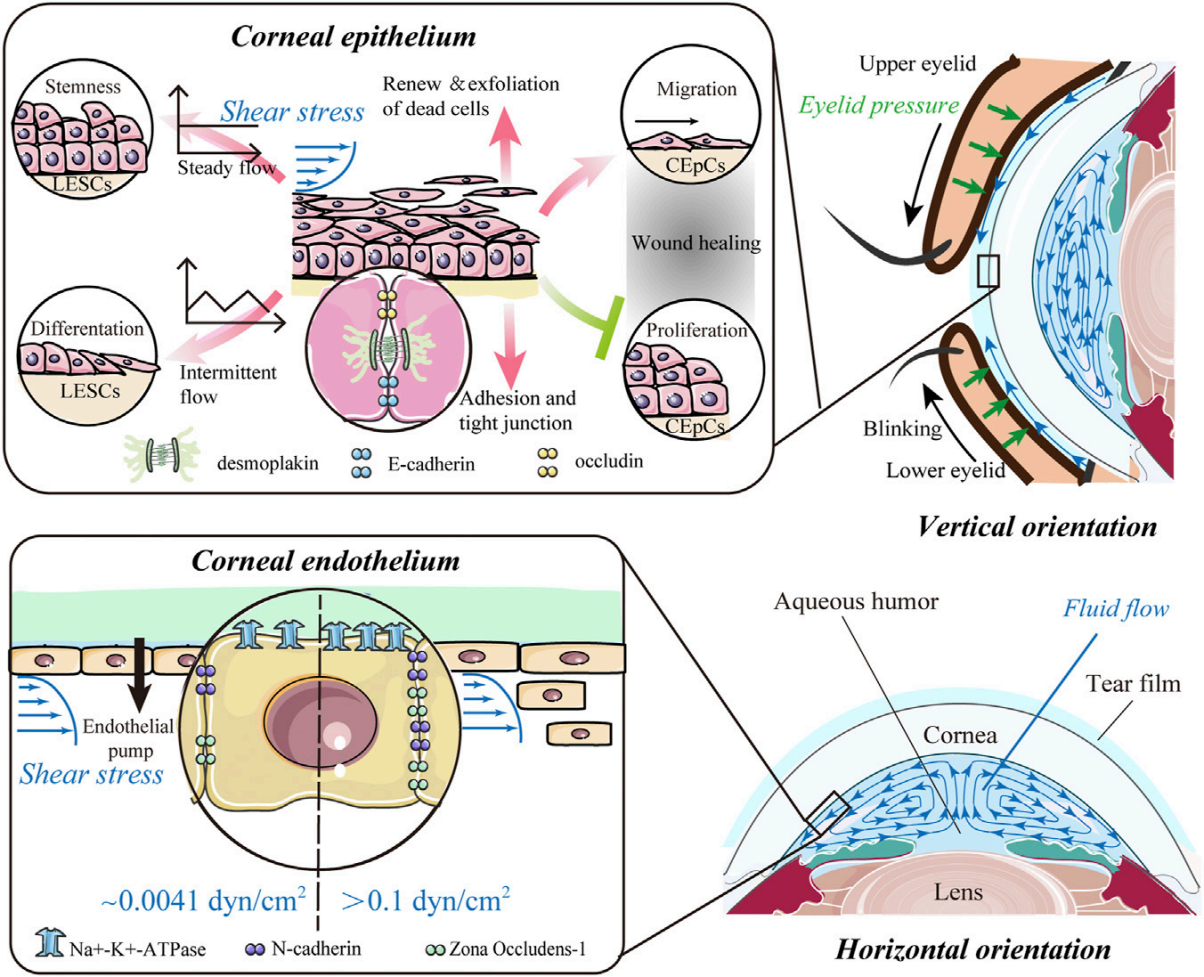
Impact of shear stress. The flow of the tear film and aqueous humor potentially results in shear stress on the anterior and posterior surfaces of the cornea and predominantly affects the epithelium and endothelium of the cornea. For corneal epithelial cells (CEpCs), steady flow maintains limbal epithelial stem cell (LESC) stemness, whereas intermittent flow induces their differentiation. Shear stress is crucial for spontaneous renewal and exfoliation of superficial epithelial cells, while it also promotes cell–cell contacts to strengthen barrier function. In the wound healing process, shear stress can mediate migration of CEpCs to facilitate wound healing, while suppressing proliferation of CEpCs to delay wound repair. Corneal endothelial cells (CEnCs) are more likely to be detached from the corneal endothelium with higher shear stress (>0.1 dyn/cm^2^). Also, the expressions of the corneal endothelium-related markers, such as ZO-1, N-cadherin, and Na^+^-K^+^-ATPase, could be upregulated with shear stress in a dose-dependent manner.

Shear stress is generated when the force is parallel to the cross-section of the material, for instance, when the fluid flows over the material surface. The flow of the tear film and aqueous humor can result in shear stress on the anterior and posterior surfaces of the cornea and mainly affects the epithelium and endothelium (Figure 2).

### The pressure and shear stress generated by eyelid motion

During spontaneous blinking, the eyelids move with respect to the ocular globe and lubricate it with a tear film to maintain a smooth epithelial surface for optical properties of the eye. The motion of the eyelid and the flux of tears also produce different types of mechanical forces over the ocular surface, especially eyelid pressure and fluid shear stress (Figure 2).

It is difficult to directly measure fluid shear stress generated by the motion of the tear film. Eyelid pressure, one of the key factors in determining fluid shear stress, was used as an alternative way to evaluate shear stress on the ocular surface (Yoshioka et al., 2015). The upper eyelid pressure (16.95 ± 6.08 mmHg–31.0 ± 6.8 mmHg) and lower eyelid pressure (16.11 ± 7.27 mmHg–29.9 ± 6.5 mmHg) measured by different approaches are summarized in Table 1 (Miller 1967; Lydon and Tait 1988; Shaw, et al., 2009; Shaw et al., 2010; Sakai et al., 2012; Namiguchi et al., 2018). The measurement using the blepharo-tensiometer showed that eyelid pressure decreased with age for both the upper and lower eyelids in healthy eyes (Sakai et al., 2012). This pressure increased in dry eye and lid-wiper epitheliopathy (Yoshioka et al., 2015; Yamamoto et al., 2016; Yamaguchi and Shiraishi 2018) but decreased in functional nasolacrimal duct obstruction (Kim, Lee, et al., 2018). This eyelid pressure is homogenously dissipated and absorbed by the corneal epithelium and conveyed to the underlying tissue. Any thinning of the epithelium and decrease in layers lead to a decrease in pressure dissipation and an increase in structural stress on each of the cells (van Setten 2020).

**TABLE 1:** Instruments to measure eyelid pressure.

Author(s)	Method	Result	Feature
Miller (1967)	Used modified contact lenses to create a chamber that was filled with water and attached to a manometer	During a blink, the average eyelid pressure was 10.3 mmHg	This system was the first method to quantify eyelid pressure *in vivo*
			The pressure is the average value of the pressure of the eyelid over a large area and the baseline changes markedly. Also, the relatively thick modified contact lenses (more than 2.5 mm) may cause distension of the eyelids and influence the accuracy of the measurements
Lydon and Tait (1988)	Used a contact lens with a silicone elastomer contact lens over the top to create a special contact lens pressure transducer	NA	The quantitative values of the eyelid pressure were not reported
Shaw et al. (2009), Shaw et al. (2010)	Used a rigid contact lens attached with a thin, (0.17 mm) piezoresistive pressure sensor to measure static upper eyelid pressure (without blinking)	The mean central upper eyelid pressure of young adults was 8.0 ± 3.4 mmHg, which was derived using the pressure-sensitive paper imprint widths	The total thickness of the device inserted between the cornea and eyelid was much smaller (less than 0.7 mm) However, the magnitudes of eyelid pressure vary when being measured by different methods	
Sakai et al. (2012), Namiguchi et al. (2018)	A thin (0.4 mm) tactile sensor was covered with silicone rubber and placed between a soft contact lens on the cornea and the inner surface of the eyelid, named as blepharo-tensiometer	The mean central eyelid pressure was 16.95 ± 6.08 mmHg to 31.0 ± 6.8 mmHg for the upper lid and 16.11 ± 7.27 mmHg to 29.9 ± 6.5 mmHg for the lower lid	The influence of intraocular pressure cannot be excluded
			The eyelid pressures under stationary conditions (i.e., eyelids closed) and dynamic conditions (i.e., during blinking) can be directly assessed with good reliability and accuracy

The levels of shear stress (and the coefficient of friction) between the cornea and eyelid are also affected by shear distribution within the tear film/mucin system and the extent to which the sliding partners make contact with each other (Pult et al., 2015). Considering these complex factors, an elastohydrodynamic mathematical model of the human eyelid wiper was developed to predict shear stresses on the ocular surface (Jones et al., 2008). However, the elastic constants or thicknesses of elastic layers, which are essential in this model, remain currently unavailable. Based on the *in vivo* tear flow turnover rate (0.31 ml/min) and a mathematical model, the magnitude of the shear stress was calculated to be 5.0 × 10^–3^ Pa (0.05 dyn/cm^2^) (Kang et al., 2014). More recently, several eye models have been proposed to mimic the interface between the ocular system and external environments (Qin et al., 2018; Phan et al., 2019; Seo et al., 2019). These advance our ability to quantitate blink-induced mechanical forces.

### Shear stress generated by aqueous humor flow

Aqueous humor (AH) is secreted by the ciliary process, passes through the pupil into the anterior chamber (AC), and drains from the eye predominantly *via* the trabecular meshwork (TM). Due to natural convection, this water-like fluid continuously circulates in the AC and produces shear stress on the corneal endothelium (lower panel in Figure 2). It is difficult to directly measure the shear stress produced by AH. Thus, several numerical calculations of AH dynamics have been developed to delineate the flow patterns and distributions of shear stress (Kumar et al., 2006; Yamamoto et al., 2010; Qin et al., 2021; Tang et al., 2022). Yamamoto et al. (2010) estimated the physiological shear stress exerted on CEnCs with an anterior chamber depth of 2.8 mm and a temperature difference between the cornea and iris of 1°C. They found that the maximum shear stress at the center of the corneal endothelial surface was 0.0062 dyn/cm^2^ (6.2 × 10^–4^ Pa), and the average shear stress was 0.0041 dyn/cm^2^ (4.1 × 10^–4^ Pa), which had little effect on CEnCs (Yamamoto et al., 2010). Based on a coupled-lattice Boltzmann model, Qin et al. (2021) estimated the average shear stress on CEnCs ranging from 1.22 × 10^–5^ Pa to 1.85 × 10^–3^ Pa in a healthy eye. The maximum shear stress was located at the center and midperiphery of the corneal endothelial surface in the standing (vertical orientation in Figure 2) and up-facing orientation (horizontal orientation in Figure 2), respectively. Moreover, shear stress in the standing orientation with a greater temperature difference across the AC was notably higher, while the inflow velocity, TM permeability, and AH viscosity have no influence on shear stress (Qin et al., 2021).

### Shear stress in the barrier function of the corneal epithelium and endothelium

The epithelium is the outer barrier of the cornea and has the highest regenerative capacity. Shear stress can regulate proliferation and differentiation of LESCs, which are crucial for renewal of epithelial cells (Figure 2). Steady flow facilitated the maintenance of LESC stemness, whereas intermittent flow induced their differentiation (Kang et al., 2014). In a model mimicking blink, the force from repetitive eyelid movement also enhanced corneal epithelial cell differentiation (Seo et al., 2019). For differentiated CEpCs, shear stress is crucial for spontaneous exfoliation of superficial epithelial cells (Figure 2). In early 1997, Ren and Wilson showed that shear stress increased the shedding rate of CEpCs and cell apoptosis (Ren and Wilson 1997). Such shear stress from blinking reached a peak at the apex of the corneal surface, which explains the increased exfoliation of dead cells preferentially from the center (Yamamoto et al., 2002). Shear flow stress also promoted cell–cell contacts between the epithelial and stromal layers (Figure 2). CEpCs under flow-induced shear stress conditions became larger, spread more, and showed more cell–cell contacts mediated by desmosomes (Hampel et al., 2018). These CEpCs formed a barrier with high expressions of cell adhesion and tight junction components, including E-cadherin, occludin, and desmoplakin, indicating strengthening of barrier function (Hampel et al., 2018; Abdalkader and Kamei 2020). Fluid shear stress also facilitated the interplay between CEpCs and fibroblasts and increased the epithelial cell layers when cultured *in vitro* (Kawata et al., 2019).

The role of shear stress in wound healing remains unclear (Figure 2). Molladavoodi et al. found that, in comparison with higher shear stress (8 dyn/cm^2^), lower shear stress (4 dyn/cm^2^) mediated more prominent, organized, and elongated filamentous actin of CEpCs to facilitate wound healing (Molladavoodi et al., 2017). However, Utsunomiya et al. reported that shear stress (1.2 dyn/cm^2^ or 12 dyn/cm^2^) could delay wound repair and suppress proliferation of CEpCs, which was associated with an increase in TGF-β1 and SMAD2 phosphorylation (Utsunomiya et al., 2016). It is to be noted that the seemingly contradictory effects on wound healing of the corneal epithelium might be due to different levels of shear stress applied in distinct contexts. It is also worth noting that the shear stresses used in these models (Utsunomiya et al., 2016; Molladavoodi et al., 2017) are far beyond the physiological range (0.05 dyn/cm^2^) and thus could not mimic the physiological ocular environment. More advanced *in vitro* models are required to better-elucidate the response of CEpCs to shear stress under physiological conditions. Thus, shear stress in the tear film–epithelial interface is essential for homeostasis of the epithelial layer, including maintaining regular turnover and promoting the interaction of CEpCs with their neighbors. However, its role in injured or diseased conditions needs to be assessed by establishing models that closely mimic genuine clinical scenarios.

Shear stress has the potential to regulate ECD and influence the water pump function of CEnCs (Kaji et al., 2005; Yamamoto et al., 2010). The magnitude required to detach CEnCs from the corneal endothelium was 0.1–10 dyn/cm^2^ (Kaji et al., 2005), which is considerably higher than the shear stress on normal corneal endothelial surfaces (Figure 2). Furthermore, the loss of CEnCs increased with shear stress in a dose- and time-dependent manner (Kaji et al., 2005). Yamamoto et al. (2010) further confirmed this finding and pointed out that CEnCs were more likely to be detached from the attached substrates under intermittent shear stress of the same magnitude. Recently, the expressions of the corneal endothelium-related markers ZO-1, N-cadherin, and Na^+^-K^+^-ATPase were found to be upregulated after exposure to shear stress from 0 to 2.0 dyn/cm^2^ (Figure 2) (Duan et al., 2021). All these findings show that shear stress is also essential for the corneal endothelium, such as an inner barrier to maintain corneal transparency and protect the stroma from edema.

## The impact of tensile and compressive stresses

Tensile or compressive stresses develop when a material is subjected to external stretching or compression, resulting in elongation or shortening of the object. As previously mentioned, the changes in corneal shape and topography are closely related to the altered position or tension of the eyelid, which indicated the existence of compressive stress from eyelid pressure (Collins et al., 2006; Collins, et al., 2006; Shaw, et al., 2009a). However, corneal shape is mainly influenced by the magnitude of intraocular pressure (IOP). As a pressure inside the eye, IOP is the primary cause to generate stretching force on the cornea under physiological conditions. ECM components in the stroma are responsible for maintenance of a normal corneal structure and are closely related to the response to tensile and compressive stresses.

### IOP results in the dome-shaped strain of the cornea

**FIGURE 3:**
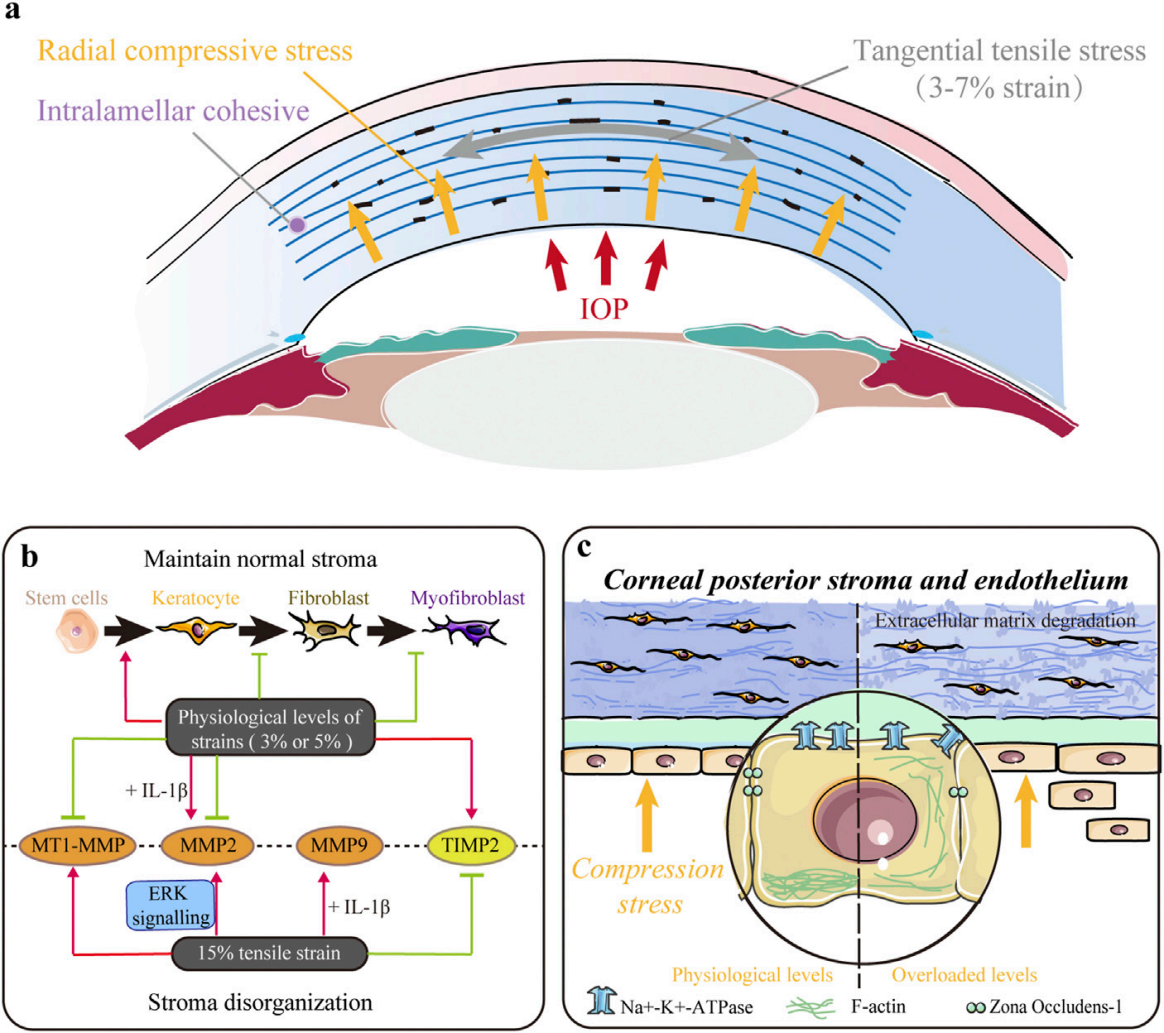
Impact of tensile and/or compressive stresses. **(A)** Native cornea is mainly subjected to tangential tensile (parallel to the plane of the cornea) stress and radial compressive stress within the entire corneal thickness. **(B)** Mechanical strains in the physiological range (∼3%) maintain a normal keratocyte phenotype, decrease keratocyte–fibroblast–myofibroblast (KFM) transformation, and inhibit the synthesis of proteases to maintain normal stromal structure. However, larger magnitude strains (∼15%) upregulate the expression of proteases and contribute to extracellular matrix (ECM) disorganization. **(C)** Mechanical compression stress indirectly controls stromal hydration and thickness by modulating the pump function of the corneal endothelium. Moreover, it also directly influences stromal structure by altering cell morphology, inhibiting proliferation, and promoting apoptosis and extracellular matrix (ECM) degradation in the stroma.

IOP is the fluid pressure within an eye and is represented by the pressure difference between the anterior chamber and atmosphere, ranging from 10 to 20 mmHg (1.33–2.66 kPa) in the normal state (Kumar et al., 2006; Liu et al., 2019). IOP induces a tangential tensile (parallel to the plane of the cornea) stress and a radial compressive stress within the entire corneal thickness (Figure 3A). The native cornea is subjected to a dome-shaped strain from IOP *in vivo* (Zhang et al., 2017). With the daily variation in IOP, the deformation of corneal shape fluctuates physiologically. In early 1977, the strains (the percentage of deformation relative to the original shape) on human corneas in the physiologic IOP range (5, 10, 25, and 45 cmH_2_O) were measured, varying from small strains (0.28%) up to 1.14% in the apical region (Shin et al., 1997). Later, Zhang et al. (2017) pointed out that the strain was within the range of 0%–3% in the stroma of the cornea.

The biomechanical properties of the cornea and its constituent materials are important in determining the IOP-induced strain. The cornea is a complex anisotropic composite (Dupps and Wilson 2006). Thus, many studies have suggested that the strain distribution varies in different regions of the cornea. In a normal cornea, the tangential stretch in the central region was significantly smaller than that in the paracentral region, and the magnitude of radial strain was significantly larger than that of tangential strain (Kwok et al., 2021). Hennighausen et al. (1998) reported that the maximal strain on the posterior surface was larger than that on the anterior surface and that the strain response to corneal swelling was reduced on the anterior surface but enhanced on the posterior surface. Hollman et al. (2002) showed that the same mechanical loading induced distinct strains in different layers of the cornea owing to differential elastic properties. These findings are consistent with the heterogeneity of the cornea in the central to peripheral, anterior to posterior, and rotational directions. In contrast, the nonlinear elastic and viscoelastic properties of the cornea are determined by the interaction between its structure, such as collagen and a polyanionic ground substance (Dupps and Wilson 2006). As such, any change in its structure in refractive surgery or other disorders can affect its biomechanical properties, which may alter the distribution of IOP-induced strain on the corneal surface (Dupps and Wilson 2006; Fang et al., 2020). This suggests that mechanical strain may be critical in maintaining the microenvironment of this tissue and the normal behavior of corneal cells.

### Tensile and compressive stresses regulate corneal stroma composition

Tensile and compressive stresses influence various behaviors of stromal cells. Acute stretching or compressive stress on the local ECM of corneal fibroblasts (CFs) changed the morphology and cytoskeleton, thus facilitating their adaption to alterations in the mechanical microenvironment through Rho and/or Rac (Petroll et al., 2004). Applying 3% equibiaxial and uniaxial strains downregulated α-SMA expression in rabbit CFs by 35% and 65%, respectively, but no difference was observed under the 15% strain (Figure 3B) (Leonard et al., 2012). Cyclic equibiaxial stretching increased cellular contractility and affected the morphology of CFs (Feng et al., 2016). Keratocytes cultured in the 3D dome-shaped model with 3% mechanical strain showed higher expressions of keratocyte markers (lumican and keratocan) and ECM components (collagen I and collagen V) than those in the flat-shaped model (Zhang et al., 2017). Interestingly, a 3% static dome-shaped mechanical strain promoted the differentiation of periodontal ligament stem cells into keratocytes (Figure 3B) (Chen et al., 2018). Therefore, mechanical strain at the physiological level may be essential in maintaining a normal keratocyte phenotype (Figure 3B).

In addition, the balance between ECM synthesis and degradation, which plays a crucial role in maintaining normal corneal structure, is disrupted by abnormal mechanical strain (Figure 3B). Matrix metalloproteinases (MMPs) are a family of proteinases that contribute to corneal ECM degradation, whereas tissue inhibitors of metalloproteinases (TIMPs) counteract proteolysis by directly binding to MMPs. Previous studies have shown that low-magnitude cyclic equibiaxial stretching (5%) alone decreased the production of MMP2 and membrane type 1 MMP (MT1-MMP) and increased the production of TIMP-2 in rabbit CFs, whereas high-magnitude stretching (15%) increased the expression of MMP2 and MMP9 in an ERK-dependent manner (Liu et al., 2014; Feng et al., 2016). Moreover, IL-1β enhanced the sensitivity of rabbit CFs to mechanical cues and increased MMP2 and MMP9 (Feng et al., 2016). Thus, mechanical strains in the physiological range (approximately 3%) decrease KFM transformation, increase the expression of collagen and proteoglycans, and inhibit the synthesis of ECM-degrading enzymes to maintain normal stromal structure. However, large-magnitude strains (15%) upregulate the expression of proteases and may contribute to ECM disorders.

Furthermore, mechanical compression interferes with the maintenance of normal stromal structures (Figure 3C). Ramirez-Garcia et al. (2018) evaluated the response of corneal endothelial cells to the indentation forces *in vitro* and found that the damage/apoptosis of CEnCs increased and ECD decreased significantly when the contact pressure exceeded 5.7 kPa (42.75 mmHg). When acute ocular hypertension (∼82.6 mmHg for 2 h) was induced, the ECD was significantly decreased, and CEnCs became irregular and multiform with disrupted ZO-1 and F-actin (Li et al., 2017). In addition, Na^+^-K^+^-ATPase was evenly distributed around the cell membrane rather than localized to the basolateral membrane (Li et al., 2017). These results indicate that compression stress indirectly controls stromal hydration and thickness by modulating the pump function of the corneal endothelium. Our latest research found that mechanical compression could also alter cell morphology, inhibit proliferation, induce apoptosis, upregulate genes related to ECM degradation, and downregulate corneal structural genes in human CFs, thus directly demonstrating the critical role of compression stress (Zhang et al., 2021).

## The association of corneal diseases with mechanical cues in the clinic

It has been demonstrated in the clinic that mechanical cues change in several primary and secondary corneal disorders, such as keratoconus, complications of contact lens use or surgical treatments, and myopia. Elucidating the influence of these abnormal mechanical cues on the functions of corneal tissue and cells will help us better understand the role of mechanobiology in corneal pathology and bridge the gap between clinical findings and basic research.

### Eye rubbing-related keratoconus

**FIGURE 4:**
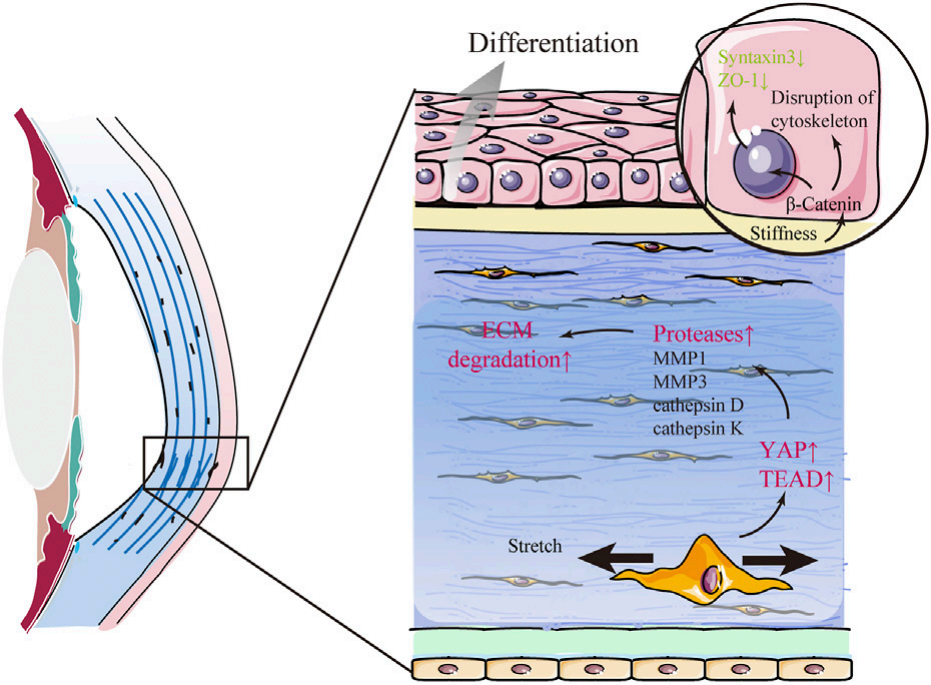
Keratoconus (KC) is related to changes in mechanical cues. In KC, β-catenin in corneal epithelial cells acts as a mechano-transducer of substrate stiffness that induces abnormal differentiation and structural changes in the corneal epithelium by delocalizing from the membrane to the cytosol. Furthermore, mechanical stretch promotes the expression of several proteases and aggravates extracellular matrix (ECM) degradation *via* YAP and its cooperator, TEA domain transcription factor (TEAD), in stromal cells.

Keratoconus (KC) is a progressive corneal ectasia characterized by a cone-shaped cornea with local thinning and weakening in the corneal stroma (Figure 4). Compared with the normal cornea, the KC cornea has marked regional heterogeneity and larger strains in the cone region (Kwok et al., 2021). In the local thinning stroma, the number of lamellae decreases. However, the compaction of collagen fibrils within individual lamellae does not change (Vellara and Patel 2015). It has been proposed that the loss of structural integrity in the KC cornea is caused by redistribution and slippage between the lamellae rather than collagen degradation (Vellara and Patel 2015). The mechanism of structural alterations in space between lamellae is yet to be elucidated. Nonetheless, it is widely considered to be related to changes in mechanical cues. Several factors play a mechanoresponsive role in KC. β-catenin in CEpCs acts as a mechano-transducer of substrate stiffness that induces structural changes, such as the disruption of the cytoskeleton (F-actin), loss of polarity (Syntaxin3), and barrier function (ZO-1), by delocalizing from the membrane to the cytosol in KC (Figure 4) (Amit et al., 2020). YAP and its cooperator, TEA domain transcription factor (TEAD), in stromal cells are the mechanotransducers of stretch that prompt protease production (including MMP1, MMP3, cathepsin D, and cathepsin K) in KC (Figure 4) (Dou et al., 2022). Some case reports have indicated that eye rubbing is closely associated with KC (Table 2) (Bawazeer et al., 2000; Weed et al., 2008; Kandarakis et al., 2011; Panahi-Bazaz et al., 2014; Gunes et al., 2015; Panikkar et al., 2016; Yusuf and Salmon, 2016; Bral and Termote 2017). Eye rubbing is a process that pushes the eyelid against the cornea with horizontal eyelid motion (Masterton and Ahearne 2018). During rubbing, various changes in mechanical cues occur, including large IOP spikes, high hydrostatic tissue pressure, and altered shear stress (McMonnies 2009; Masterton and Ahearne 2018). Vigorous rubbing may increase IOP to more than ten times its normal level (McMonnies 2008), and KC eyes may experience more significant changes in IOP than healthy eyes (Henriquez et al., 2019). Importantly, corneal hysteresis (CH) and corneal resistance factor (CRF), which are used to measure corneal biomechanical properties, were significantly lower after eye rubbing in both keratoconic and healthy eyes (Liu et al., 2011; Henriquez et al., 2019). Nevertheless, many cases of eye-rubbing-related KC remain poorly understood. Standardized clinical analysis to describe the direction, frequency, and magnitude of eye rubbing in individuals is still lacking (Prakasam et al., 2012; Balasubramanian et al., 2013). Previous studies focused on the effect of single-cycle rubbing instead of the long-term effect of multiple rounds of rubbing (Greiner et al., 1985; Greiner et al., 1997). Thus, more rigorous investigations are required in future to demonstrate the causal or other roles of eye rubbing or rubbing forces in KC development.

**TABLE 2:** Case reports on eye rubbing-related keratoconus.

Author(s)	Method	Sample size	Finding
Weed et al. (2008)	Prospective observational study	*n* = 200	Forty-eight percent of subjects reported significant eye rubbing, and there was a statistically significant difference (two samples *t*-test *p* = 0.018) between keratoconus and control groups
Bawazeer et al. (2000)	Case-control study	*n* = 120	The most significant cause of keratoconus is eye rubbing. Atopy may contribute to keratoconus but most probably *via* eye rubbing associated with the itch of atopy
Bral and Termote, (2017)	Case report	*n* = 1	Unilateral keratoconus described in a patient with the medical history revealed a habit of chronic eye rubbing only in one eye
Yusuf and Salmon (2016)	Case report	*n* = 1	Keratoconus is described in a patient with obsessive–compulsive eye rubbing in the periocular contact dermatitis and allergic eye disease
Panikkar et al. (2016)	Case report	*n* = 1	Keratoconus described in a patient with obsessive–compulsive eye rubbing
Gunes et al. (2015)	Case report	*n* = 1	Keratoconus described in a 4-year-old patient with obsessive–compulsive eye rubbing
Panahi-Bazaz et al. (2014)	Case report	*n* = 1	Keratoconus described in a 7-year-old patient with obsessive–compulsive eye rubbing
Kandarakis et al. (2011)	Case report	*n* = 1	Keratoconus is described in a patient with obsessive–compulsive eye rubbing in the context of Tourette syndrome

### Homeostasis of the ocular surface

Dry eye disease (DED) is a disease characterized by loss of homeostasis of the tear film (Craig et al., 2017). A sufficient lubrication film is essential for reducing the shear stress by preventing solid-to-solid contact between the eyelid wiper and ocular surface (Jones et al., 2008). The mean eyelid pressure in DED is approximately 1.25 times greater than that in normal eyes, indicating that higher incidence of DED and ocular surface damage are associated with higher pressure of the lids onto the ocular surface (Yoshioka et al., 2015; Yamaguchi and Shiraishi 2018). In addition, the impairment of lubrication in DED also increases shear stress by decreasing the separation between the eyelid and ocular surface (Van Setten 2020). Hence, DED may affect cell behavior by altering mechanical cues from the tear film and ocular surface.

A contact lens (CL) is an ocular prosthetic device used for vision correction that can also change the mechanical microenvironment of the ocular surface. As eyelids move across the ocular surface during blinking, it creates a mechanical force on the CL, causing it to move laterally (up–down) and transversally (in–out) (Chauhan and Radke 2001; Pult et al., 2015). This force generated on the CL is transferred onto the cornea and causes greater corneal deformation (Ramasubramanian et al., 2022). In general, the force is reduced as CL approaches the inferior cornea and induces less mechanical trauma at the inferior cornea and limbus (Ramasubramanian et al., 2022). On the other hand, CL can directly restrict tear flow over the corneal surface (Mann and Tighe 2013; Muntz et al., 2015), which reduces shear stress where the epithelium is usually subjected and protects epithelial cells from blinking-induced shear force (Yamamoto et al., 2002). However, the movement and deformation of the CL during blinking can also induce tear flow that creates shear stress on the epithelium (Chauhan and Radke 2001; Pult et al., 2015). In addition, the mechanical force imposed on the cornea by a specially designed reverse-geometry gas-permeable rigid CL, the orthokeratology (OK) lens, is expected to be higher than that imposed by a routine CL (Ding et al., 2012). This external force placed against the front surface of the cornea may modify or eliminate the refractive error by reshaping the cornea with thinning of the central part and thickening of the paracentral corneal epithelium (Swarbrick 2004; Li et al., 2016; Chen et al., 2017; Kim WookKyum et al., 2018). In addition, wearing an OK lens reduced CH and CRF (Lam et al., 2019). However, the cellular mechanism for this subtle remodeling of the anterior corneal layers remains limited, and the impact of this external force on the corneal tissue at the microscopic level or cell activities also remains obscure (Swarbrick 2004; Ding et al., 2012).

### The progression of myopia

Myopia is one of the most common ocular problems, affecting approximately 22% of the current world population; however, the exact cause of myopia is complicated and remains unclear. Recent studies have focused on the changes in corneal biomechanical properties in myopia. In a meta-analysis of corneal biomechanical properties of 11 related studies using an ocular response analyzer, we found that corneal elasticity decreased significantly in high myopia (Wu et al., 2019). We further showed that corneal stiffness of over 1,000 patients with high myopia provided by Corvis ST also significantly reduced (Han et al., 2020), which is further supported by the findings from other groups (Zhang et al., 2018; Asano et al., 2019; Long et al., 2019; Kenia et al., 2020; Sedaghat et al., 2020; Tubtimthong et al., 2020). With an experimentally induced myopia model in chicks, the reduction of corneal elasticity and weakness of corneal biomechanics were related to development of myopia (Kang et al., 2018). Using an atomic force microscope, we recently observed that the stiffness of single cells harvested from the cornea in chicks with high myopia reduced and then returned to normal after the vision was resumed (Xin et al., 2021). However, to date, it remains unclear whether myopia causes these biomechanical changes in the corneal tissue and cells or vice versa.

### Surgical intervention disturbs the balanced mechanical microenvironment

Corneal refractive surgery, such as small-incision lenticular extraction (SMILE), laser-assisted *in situ* keratomileusis (LASIK), and photorefractive keratectomy (PRK), is the most common method for correcting a refractive error by central ablation to remodel the corneal surface structure and curvature. During the surgical procedure and postoperative recovery, different types of mechanical cues change in the corneal microenvironment. IOP fluctuates considerably over time during surgery. The mean IOP measured by an infusion cannula inserted through the limbus was lower in corneal flaps when the surgery was conducted by a femtosecond laser than that by a microkeratome during globe suction (81.78 vs. 122.51 mm Hg) and cutting (62.25 vs. 141.02 mm Hg) (Chaurasia et al., 2010). Our previous study monitored the intraoperative IOP during SMILE surgery and showed that IOP significantly increased after suction initiation (up to 86.55 ± 22.36 mmHg) and was stabilized at the cutting step (up to 75.87 ± 23.17 mmHg) (Cheng et al., 2018). These IOP fluctuations during corneal refractive surgery may contribute to complications in retinal function (Charteris et al., 1997; Qin et al., 2007). However, it remains unclear whether and how corneal cells respond to such a sharp increase in IOP. On the other hand, several forces, including the negative intrastromal fluid pressure generated by the hydrophilia of stromal glycosaminoglycans, cohesive forces between lamellae, centripetal force, and the lamellar tension manifested by the IOP, contribute to the corneal steady state and undergo complex disruptions during corneal refractive surgery (Dupps and Wilson 2006). Thus, corneal cells sense and respond to these mechanical alterations and remodel such areas to restore mechanical integrity (Dupps and Wilson 2006). Furthermore, understanding of these processes will improve the predictability of refractive surgery and minimize complications such as refractive regression or keratectasia.

Laser iridotomy (LI) is a commonly used treatment for glaucoma. The number of cases of bullous keratopathy after LI has increased over the years (Lim et al., 2006; Ang et al., 2007). The shock wave of the laser, increased temperature of aqueous humor, and changes of cytokines in AH cannot fully explain why bullous keratopathy often develops even years after LI. It has been speculated that hydrodynamic changes in aqueous flow might play a key role in LI-induced bullous keratopathy (Kaji et al., 2005; Yamamoto et al., 2010). Yamamoto et al. (2006) showed in an animal study that during miosis, the AH was ejected into the anterior chamber from the posterior chamber through the LI window to strike the corneal endothelium, while the AH flowed oppositely during mydriasis (Yamamoto et al., 2006). Abnormal aqueous flow might result in excessive tensile and shear stresses on the CEnCs (Yamamoto et al., 2006; Yamamoto et al., 2010). Kaji et al. (2005) postulated a virtual model of LI for analysis and reported a maximum shear stress up to 1 dyn/cm^2^, which was hundred-fold higher than the stress under the physiological state and may facilitate development of bullous keratopathy. Yamamoto et al. (2010) used a computational fluid dynamics model to compare shear stress with varied anterior chamber depths and found that the shear stress was 70-fold greater than that under the physiological state when the anterior chamber depth was 1.0 mm (Yamamoto et al., 2010). As a result, patients with insufficient anterior chamber depth after LI may suffer from excessive shear stress caused by the flow of AH through the LI window, leading to a high risk of CEnC damage and loss (Jin and Anderson 1990; He et al., 2007). To understand and avoid these complications involved in excessive mechanical stimuli or structural alterations due to surgical intervention, it is necessary to take into account the mechanical changes of the corneal microenvironment during and after surgery.

## Conclusion

Mechanical forces are involved in many aspects of both the physiology and pathology of the cornea and have profound influences on corneal cells. Growing evidence suggests that mechanical factors play an important role in development and progression of various diseases. Moreover, mechanical cues can mediate the differentiation capacity of tissue-specific stem cells. However, the specific effect of mechanical forces at the corneal embryonic stage and the relationship between the changes in mechanical cues and corneal disorders remains unclear. This underscores the urgent need to assess specific mechanotransduction pathways and signals in corneal cells. It is likely that mechanotransduction and biochemical signaling are intertwined, which synergistically influences cellular functions of corneal cells and the mechanical microenvironment. Therefore, elucidating the influence of these mechanical cues on the functions of corneal tissue will help us better understand the mechanisms underlying corneal diseases and facilitate development of novel therapeutic strategies against these diseases from the perspective of biomechanics and mechanobiology.
